# Multilevel Analysis of Glutathione-Induced Responses in Pepper Seedlings Under Water-Deficit Conditions

**DOI:** 10.3390/plants15182831

**Published:** 2026-09-16

**Authors:** Melek Ekinci, Esma Yigider, Metin Turan, Selda Ors, Murat Aydin, Ertan Yildirim

**Affiliations:** 1 Department of Horticulture, Faculty of Agriculture, Atatürk University, Erzurum 25240, Türkiye; ekincim@atauni.edu.tr; 2 Department of Agricultural Biotechnology, Faculty of Agriculture, Atatürk University, Erzurum 25240, Türkiye; esma.yigider@atauni.edu.tr (E.Y.); maydin@atauni.edu.tr (M.A.); 3 Department of Agricultural Trade and Management, Business School, Yeditepe University, Istanbul 34755, Türkiye; metin.turan@yeditepe.edu.tr; 4 Department of Agricultural Structures and Irrigation, Faculty of Agriculture, Atatürk University, Erzurum 25240, Türkiye; seldaors@atauni.edu.tr

**Keywords:** *Capsicum annuum* L., glutathione, drought, antioxidant system, biostimulant

## Abstract

Drought stress is a major abiotic factor that negatively affects plant growth, physiological processes, and yield in pepper cultivation. This study evaluated the ameliorative effects of exogenous glutathione (GSH) at 50 µM and 100 µM in pepper seedlings grown under water-restricted conditions (60% field capacity), using morphological, physiological, and biochemical parameters. Water restriction significantly reduced seedling height, stem diameter, leaf area, and plant and root fresh and dry weights, and decreased chlorophyll a, chlorophyll b, total chlorophyll content, and plant growth hormones. Conversely, water restriction significantly increased hydrogen peroxide (H_2_O_2_), malondialdehyde (MDA), proline, sucrose, and abscisic acid (ABA) content. Glutathione application, on the other hand, significantly reduced water-restriction-induced growth damage, increased biomass production and chlorophyll content, and supported the antioxidant defense capacity of plants by decreasing H_2_O_2_ and MDA levels, which are indicators of oxidative stress. It also improved osmotic balance by regulating proline and sucrose accumulation and increased seedling adaptation to water restriction. In conclusion, exogenous GSH acted as a potential biostimulant under water-deficit conditions, with its beneficial effects associated with improved osmotic adjustment through the regulation of proline and sucrose accumulation, enhanced antioxidant defense, and modulation of endogenous phytohormone levels.

## 1. Introduction

Drought is a water deficiency resulting from a sustained decrease in precipitation and high evaporation rates, leading to insufficient moisture for normal plant growth and development [1]. Globally, drought is considered one of the most destructive factors reducing crop production, surpassing other environmental stressors, and poses the most serious threat to global food security and sustainable agriculture [2,3]. With global climate change, it is estimated that 50% of arable land could be affected by drought stress by 2050. Drought, which can cause losses of up to 70% depending on crop species, leads to economic damage as well as loss of agricultural land, desertification, ecosystem degradation, and food shortages, significantly impacting human health [4,5,6,7,8,9].

Drought is a complex, multifaceted stress that affects plants at the morphological, physiological, biochemical, and molecular levels [1,10]. Water deficiency causes turgor loss and inhibits cell expansion, leading to reduced plant height, smaller leaf area, leaf curling, premature senescence, and chlorosis. Plants may increase their root-to-stem ratio to optimize water uptake [2,5,7]. To conserve water, plants close their stomata and restrict CO2 uptake, reducing photosynthetic capacity, slowing transpiration, and decreasing leaf relative water content and water potential [1,11,12]. Excessive accumulation of reactive oxygen species (ROS) in cells causes oxidative stress and damage to proteins, lipids, and DNA. In response, osmoprotectants such as proline, glycine betaine, and sugars accumulate, enhancing osmotic regulation and antioxidant enzyme activity [3,13,14]. These effects impair seed germination, seedling development, flowering, fruit set, and ripening, reducing plant growth, development, and yield [5,6,7].

Glutathione (GSH; γ-glutamyl-cysteinyl-glycine) is an important tripeptide thiol compound that is widely found in plant tissues, is low molecular weight, water-soluble, and non-protein [15,16]. It plays a role in the plant defense system as a direct antioxidant and vital signaling molecule in cellular processes [17,18]. GSH is composed of the amino acid glutamate, cysteine, and glycine [19]. The bond between glutamate and cysteine is formed via a γ-carboxyl group, and this bond provides high stability by protecting the molecule from degradation by aminopeptidases [15,16]. Its biosynthesis involves a two-stage ATP-dependent process. In the first stage, the γ-glutamylcysteine synthetase (γ-ECS) enzyme, which is the rate-limiting step of the synthesis, combines glutamate and cysteine to form γ-glutamylcysteine [17,20]. In the second step, the GSH synthetase (GSHS or GS) enzyme synthesizes the final GSH molecule by adding glycine to γ-glutamylcysteine [19,20]. The fundamental mechanism of action of GSH is based on the redox (reduction–oxidation) capacity of its sulfhydryl (-SH) group [18]. GSH, as a key component of the ascorbate–glutathione cycle, provides electrons to convert oxidized ascorbate (DHA) back to its reduced form (AsA), and in the process, it itself is oxidized to the GSSG form [18,19,20,21]. It is the most important determinant of cellular redox status, and the GSH reductase (GR) enzyme reduces GSSG back to GSH using NADPH and maintains this balance [18,19,20]. It conjugates herbicides and xenobiotics through the glutathione S-transferase (GST) enzyme, trapping or inactivating them in the vacuole [15,18]. It plays a role in cell division, root meristem function, embryo development, and the regulation of flowering time [17,22]. It also has significant effects on plant stress tolerance. GSH has several functions in stressed plants, including directly scavenging ROS and coordinating stress tolerance as a signaling agent that regulates gene expression and metabolic processes [15,22]. In fact, exogenous GSH is used to mitigate the negative effects of various environmental stressors (drought, salinity, heavy metals, extreme temperatures, etc.), and exogenous GSH application promotes plant growth, maintains photosynthetic capacity, and stabilizes cellular redox balance.

GSH directly chelates certain metal ions and also serves as the precursor for phytochelatein biosynthesis [19,23]. GSH also contributes to plant tolerance to other abiotic stresses in which oxidative stress is a major component of cellular damage. Exogenous GSH has been reported to alleviate heavy metal toxicity, including Pb and Cr stress, by enhancing antioxidant defense and reducing oxidative damage [23,24]. Recent evidence further demonstrates that Pb stress is associated with changes in stomatal responses, oxidative stress, and antioxidative defense, highlighting the central role of redox regulation in plant responses to abiotic stress [25]. These findings support the broader role of oxidative stress management in stress tolerance and provide a physiological basis for investigating GSH as a potential mitigator of water-deficit stress in pepper. It is an important cofactor in the detoxification of methylglyoxal (MG), a toxic compound in stress [26]. MG is converted to D-lactate via the glyoxalase system (Gly I and Gly II enzymes) where GSH is used [15,26]. During drought, GSH is effective in regulating stomal conductance, maintaining tissue water content (RWC), and balancing plant water loss by increasing the levels of osmoprotectants such as proline [27,28]. GSH is effective in reducing heat-induced lipid peroxidation (MDA) and preventing chlorophyll degradation, thereby preserving photosynthetic capacity [18,28]. Furthermore, it helps maintain chloroplast membrane fluidity and stabilize antioxidant enzyme activities at low temperatures, thereby reducing cold damage [21,29].

Peppers are among the most widely produced and consumed vegetable species worldwide. Regular irrigation is crucial in their cultivation. Insufficient irrigation negatively affects all processes, from plant growth and development to fruit set and ripening. There is a lack of sufficient studies on the effect of exogenous GSH application on peppers under drought stress. Glutathione was selected in the present study as a potential biostimulant for mitigating water-deficit stress because of its multifunctional role in plant redox regulation and stress adaptation. In addition to its antioxidant function, GSH is involved in osmotic adjustment and in regulating physiological and hormonal responses under adverse environmental conditions. We hypothesized that exogenous GSH application would alleviate the adverse effects of water deficit in pepper seedlings by maintaining plant growth and photosynthetic pigments, reducing oxidative stress, enhancing antioxidant defense and osmotic adjustment, and modulating endogenous phytohormone levels. Therefore, this study evaluated the effects of 50 and 100 µM GSH on morphological, physiological, biochemical, antioxidant, and hormonal responses of pepper seedlings under water-deficit conditions.

## 2. Results

According to the two-way ANOVA results, water regime (WR) and GSH application significantly affected most growth and morphological characteristics (Table 1). Specifically, the individual effects of WR and GSH were significant for plant height (PH), stem diameter (SD), leaf area (LA), plant and root fresh weights (PFW and RFW), and plant and root dry weights (PDW and RDW) (*p* < 0.001). However, chlorophyll content (CC-SPAD) was not statistically affected by the applications (Table 1). A significant interaction was observed between WR and GSH for SD (*p* < 0.05), LA (*p* < 0.001), PFW (*p* < 0.001), RFW (*p* < 0.001), PDW (*p* < 0.05), and RDW (*p* < 0.001). However, the interaction effect on PH was not significant (Table 1).

**Figure 1:**
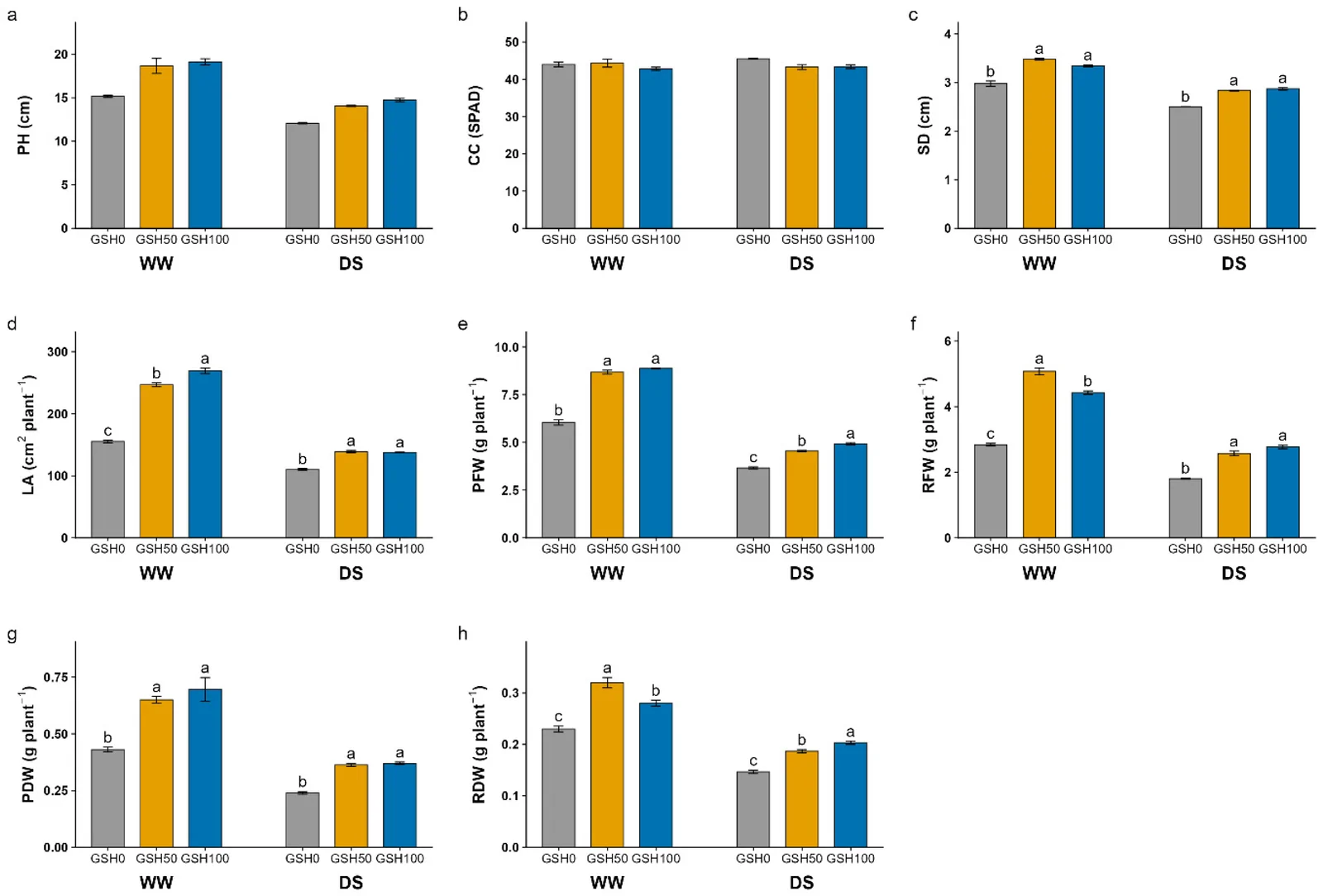
Effects of glutathione (GSH) application on growth and biomass characteristics of pepper plants under well-watered (WW) and drought stress (DS) conditions. (**a**) plant height (PH), (**b**) chlorophyll content (CC, SPAD), (**c**) stem diameter (SD), (**d**) leaf area (LA), (**e**) plant fresh weight (PFW), (**f**) root fresh weight (RFW), (**g**) plant dry weight (PDW), and (**h**) root dry weight (RDW). Bars represent the mean ± standard error (SE). Different lowercase letters above the bars indicate statistically significant differences among GSH treatments within the same water regime (WW or DS) according to Tukey’s multiple comparison test (*p* < 0.05).

**Figure 2:**
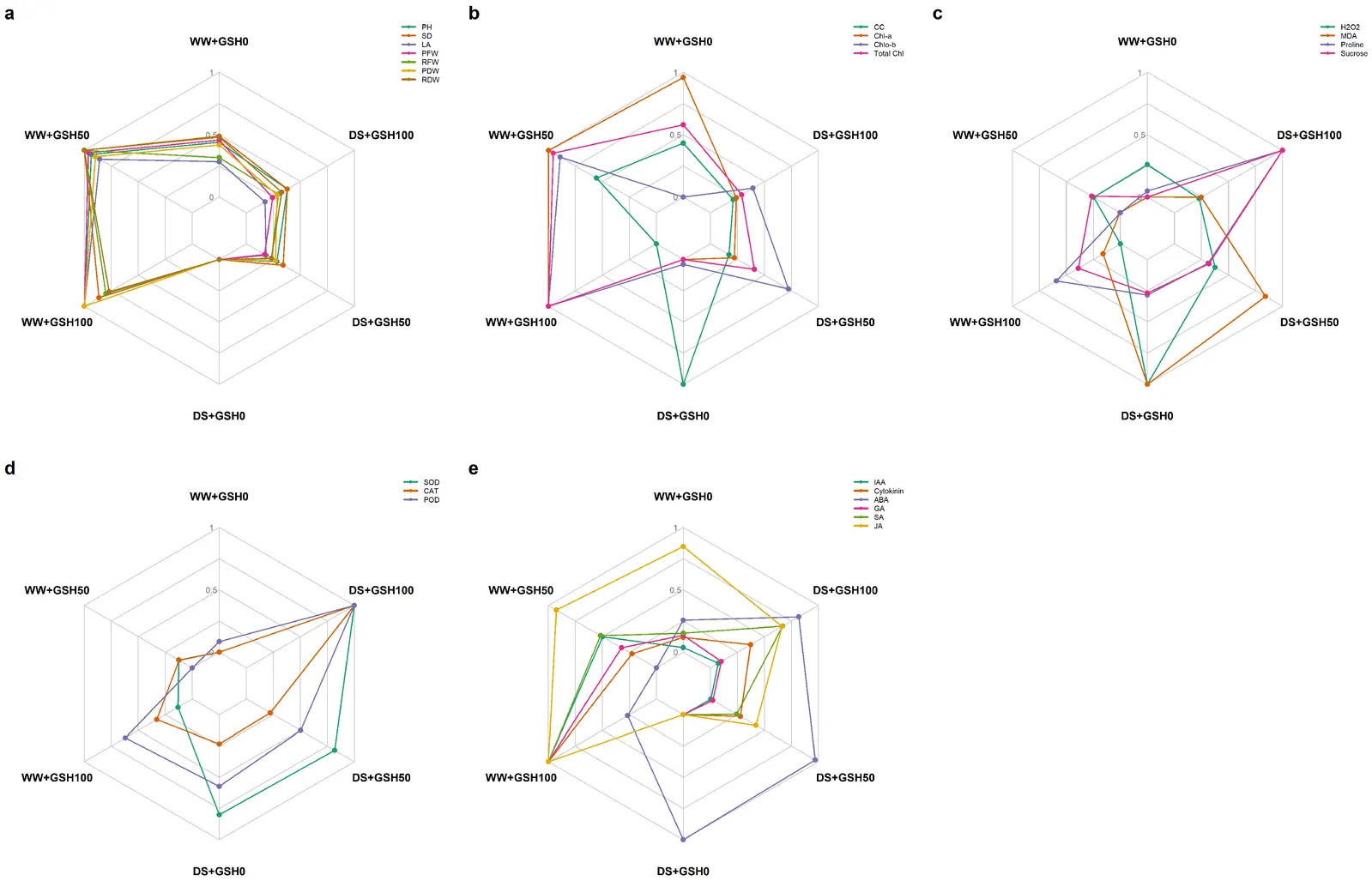
Radar charts illustrating the effects of glutathione (GSH) application on the relative responses of measured parameters in pepper plants under well-watered (WW) and drought stress (DS) conditions. (**a**) growth and biomass traits, (**b**) photosynthetic pigments (**c**) oxidative stress markers and osmolytes, (**d**) antioxidant enzyme activities, and (**e**) phytohormone levels. The abbreviations of the analyzed variables are provided in Table 1.

PH, SD, and LA were significantly inhibited under drought stress (DS) but markedly improved with exogenous GSH application (Figure 1a,c,d). Under adequate irrigation (WW), both PH and LA consistently increased with high GSH doses. Compared to the GSH-free control (WW+GSH0; PH: 15.17 cm; LA: 155.43 cm^2^ plant^−1^), the 100 µM GSH application (WW+GSH100) yielded the highest values (PH: 19.13 cm; LA: 269.58 cm^2^ plant^−1^), representing increases of approximately 26% and 73%, respectively (Figure 1a,d). Stem diameter was highest in WW+GSH50 (3.48 cm) (Figure 1c). Shoot and root biomass were highest in WW+GSH100 (Figure 1e–h and Figure 2a). 100 µM GSH increased PFW from 6.05 to 8.88 g (~47%) and PDW from 0.43 to 0.70 g (~63%) per plant compared to untreated controls (Figure 1e,g). Under drought conditions, all vegetative parameters were lowest in DS+GSH0, but GSH supplementation mitigated these reductions (Figure 1a–h and Figure 2a). PH and LA per plant decreased to 12.07 cm and 110.17 cm^2^, respectively, in DS+GSH0. PH increased by approximately 22% with 100 µM GSH (DS+GSH100). LA was significantly higher at 137.75 cm^2^ per plant with 100 µM GSH (DS+GSH100) (Figure 1a,d). Stem diameter also increased by approximately 15% in DS+GSH100 compared to DS+GSH0 (Figure 1c). Biomass accumulation was lowest in DS+GSH0, but GSH provided a significant increase, as shown in the extended radar profiles (Figure 1e–h and Figure 2a). PFW and PDW increased by approximately 35% and 54%, respectively, in DS+GSH100, while RFW and RDW were approximately 54% and 39% higher compared to DS+GSH0 (Figure 1e–h). Overall, the growth and biomass results (Figure 1a–h), together with the comprehensive view obtained from the radar graphs, show that foliar application of GSH (especially at 100 µM) significantly increased the developmental vigor of pepper plants (Figure 1a–h and Figure 2a and Table 1). The large increase in radar field at DS+GSH100, compared to the highly mitigating effect of DS+GSH0, demonstrates that GSH is significantly effective in reducing drought-induced growth suppression and supporting biomass stability (Figure 1a–h and Figure 2a).

**Figure 3:**
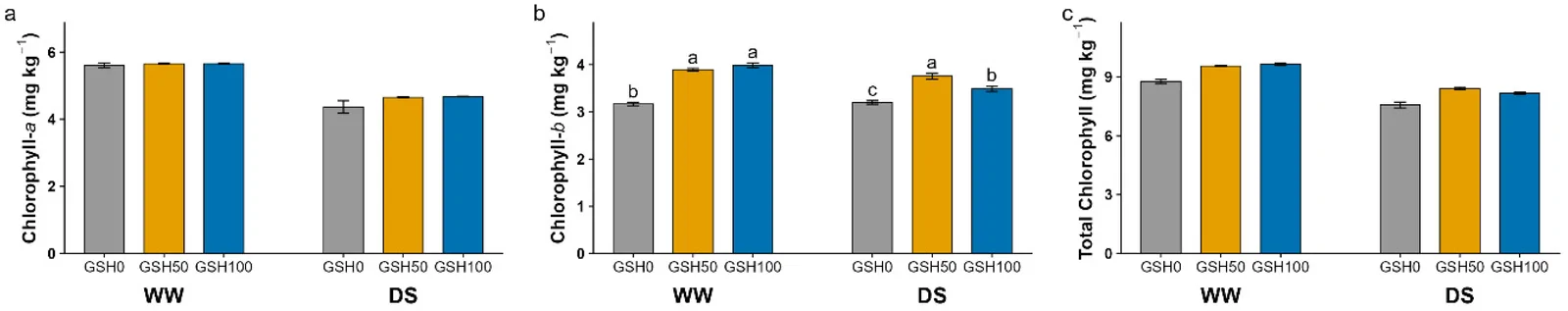
Effects of glutathione (GSH) application on photosynthetic pigments of pepper plants under well-watered (WW) and drought stress (DS) conditions. (**a**) chlorophyll a, (**b**) chlorophyll b, and (**c**) total chlorophyll. Bars represent the mean ± standard error (SE). Different lowercase letters above the bars indicate statistically significant differences among GSH treatments within the same water regime (WW or DS) according to Tukey’s multiple comparison test (*p* < 0.05).

According to the two-way ANOVA results, WR significantly affected chlorophyll a, chlorophyll b, and total chlorophyll content (*p* < 0.001). GSH application had a significant effect on chlorophyll b and total chlorophyll (*p* < 0.001), but its effect on chlorophyll a was not statistically significant. A significant WR × GSH interaction was found specifically for chlorophyll b (*p* < 0.001), while the interaction did not significantly affect chlorophyll a and total chlorophyll content (Table 1). Drought stress significantly reduced pigment content, but exogenous GSH increased it in both water regimes (Figure 3a–c). Under WW conditions, the highest pigment levels occurred in WW+GSH100. Compared to WW+GSH0, 100 µM GSH increased chlorophyll b by 25.78% (to 3.98 mg g^−1^) and total chlorophyll by 9.92% (to 9.64 mg g^−1^) (Figure 3b,c). Chlorophyll a was also highest at WW+GSH100 (5.66 mg g^−1^), but its increase was only 0.96% compared to WW+GSH0 (Figure 3a). In DS plants, the lowest levels of significantly reduced pigment content were observed at DS+GSH0 (Figure 3a,c). GSH treatment increased chlorophyll content; in DS+GSH50, chlorophyll b reached 3.76 mg g^−1^ (+17.43%) and total chlorophyll reached 8.41 mg g^−1^ (+11.16%) (Figure 3b,c). In DS+GSH100 treatment, chlorophyll a and total chlorophyll reached 4.68 mg g^−1^ and 8.16 mg g^−1^, respectively, showing increases of 7.10% and 7.92% compared to DS+GSH0 treatment (Figure 3a–c). Radar graphs further confirmed these patterns, with wider radar fields in WW+GSH100 and DS+GSH50/GSH100 treatments indicating increased pigment levels (Figure 2b and Figure 3a–c).

**Figure 4:**
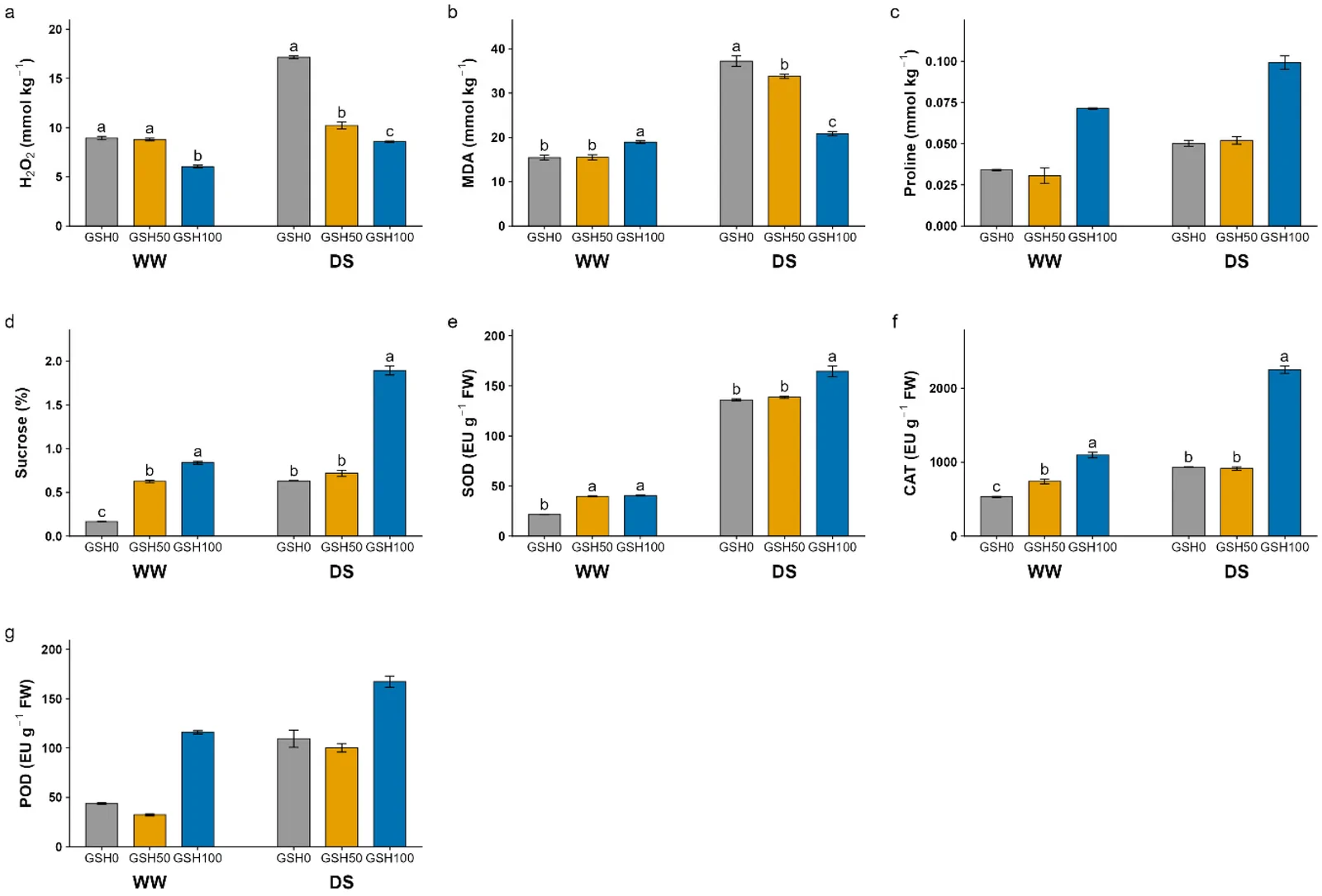
Effects of glutathione (GSH) application on oxidative stress markers, osmolyte accumulation, and antioxidant enzyme activities in pepper plants under well-watered (WW) and drought stress (DS) conditions. (**a**) hydrogen peroxide (H_2_O_2_), (**b**) malondialdehyde (MDA), (**c**) proline, (**d**) sucrose, (**e**) superoxide dismutase (SOD), (**f**) catalase (CAT), and (**g**) peroxidase (POD). Bars represent the mean ± standard error (SE). Different lowercase letters above the bars indicate statistically significant differences among GSH treatments within the same water regime (WW or DS) according to Tukey’s multiple comparison test (*p* < 0.05).

According to the two-way ANOVA results, WR, GSH, and their interaction (WR × GSH) showed significant effects (*p* < 0.001) on hydrogen peroxide (H_2_O_2_) and malondialdehyde (MDA) (Table 1). Under WW conditions, 100 µM GSH reduced H_2_O_2_ levels from 8.93 mmol kg^−1^ in WW+GSH0 to 6.04 mmol kg^−1^ in WW+GSH100, representing a 32.41% decrease. MDA, on the other hand, slightly increased from 15.44 to 18.93 mmol kg^−1^ under the same treatments (Figure 4a). Under DS, oxidative stress markers were highest in untreated DS+GSH0, with H_2_O_2_ (17.14 mmol kg^−1^) and MDA (37.22 mmol kg^−1^) values. GSH supplementation significantly mitigated this damage, reducing H_2_O_2_ and MDA levels in DS+GSH100 to 8.57 and 20.85 mmol kg^−1^, respectively, representing a decrease of approximately 50% and 44% compared to DS+GSH0 (Figure 4a,b).

The accumulation of proline and sucrose was significantly affected by both WR and GSH (*p* < 0.001). A significant interaction was observed between WR and GSH for sucrose (Table 1). Under WW conditions, proline levels increased from 0.04 mmol kg^−1^ in WW+GSH0 to 0.07 mmol kg^−1^ in WW+GSH100, showing an increase of 109.87%. Similarly, sucrose levels increased from 0.17% to 0.84%, showing an increase of 406.20% (Figure 4c,d). This stimulating effect intensified under DS. Proline and sucrose in DS+GSH100 reached 0.10 mmol kg^−1^ and 1.90%, respectively, showing increases of 97.55% and 200.30% compared to DS+GSH0 (Figure 4c,d).

GSH significantly increased the activities of antioxidant enzymes SOD, CAT, and POD under both WW and DS conditions (*p* < 0.001), with marked WR × GSH interactions observed for SOD and CAT (Table 1). Under irrigation conditions, 100 µM GSH increased the activities of SOD, CAT, and POD by 87.60%, 106.51%, and 164.57%, respectively (Figure 4e–g). While DS alone increased primary antioxidant activity, GSH provided an additional significant increase. In DS+GSH100, SOD, CAT, and POD activities reached 164.56, 2252.44, and 167.17 EU g^−1^ FW, respectively, showing increases of 20.99%, 141.72%, and 52.73% compared to DS+GSH0 (Figure 4e–g).

Integrated radar field analysis confirmed these findings, demonstrating that GSH’s suppression of oxidative markers, along with a simultaneous increase in osmoprotective and antioxidant enzyme systems, contributed to higher physiological resilience (Figure 2c,d and Figure 4a–g). The significant broadening of the radar field in DS+GSH100 compared to DS+GSH0 highlights that the 100 µM dose effectively enhances stress tolerance and maintains cellular homeostasis under water-deficient conditions (Figure 2c,d and Figure 4a–g).

ANOVA results showed that WR, GSH administration, and their interaction (WR × GSH) had highly significant effects on the endogenous levels of all measured phytohormones, including indole-3-acetic acid (IAA), cytokinin, abscisic acid (ABA), gibberellic acid (GA), salicylic acid (SA), and jasmonic acid (JA) (Table 1).

**Figure 5:**
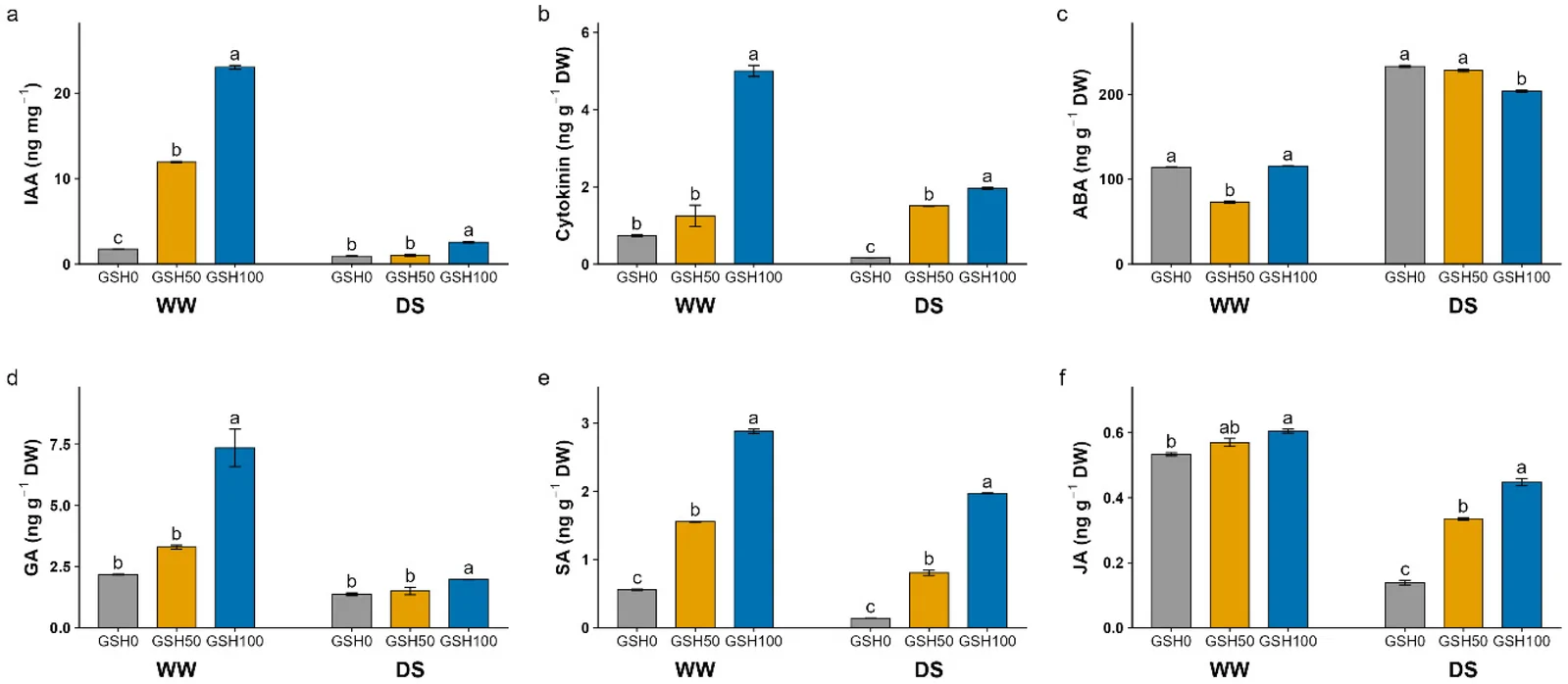
Effects of glutathione (GSH) application on endogenous phytohormone levels in pepper plants under well-watered (WW) and drought stress (DS) conditions. (**a**) indole-3-acetic acid (IAA), (**b**) cytokinin, (**c**) abscisic acid (ABA), (**d**) gibberellic acid (GA), (**e**) salicylic acid (SA), and (**f**) jasmonic acid (JA). Bars represent the mean ± standard error (SE). Different lowercase letters above the bars indicate statistically significant differences among GSH treatments within the same water regime (WW or DS) according to Tukey’s multiple comparison test (*p* < 0.05).

Under WW conditions, GSH application significantly increased growth-related hormone levels. IAA levels increased from 1.75 ng g^−1^ in the control group (GSH0) to 11.93 ng g^−1^ with 50 µM GSH and to 23.03 ng g^−1^ with 100 µM GSH, showing increases of 580.95% and 1214.85%, respectively (Figure 5a,b). Cytokinin levels similarly increased from 0.74 ng g^−1^ DW in the WW+GSH0 group to 5.00 ng g^−1^ DW in the WW+GSH100 group, showing an increase of 580.72% (Figure 5a,b). Drought stress (DS) caused a significant decrease in these hormones, with cytokinin levels falling to 0.16 ng g^−1^ dry weight in untreated DS+GSH0 plants. However, GSH supplementation led to a significant improvement; in the DS+GSH100 group, IAA and cytokinin levels increased to 2.55 ng g^−1^ and 1.96 ng g^−1^ dry weight, respectively, representing increases of 173.95% and 1135.85% compared to the untreated drought control (Figure 5a,b).

ABA levels were significantly affected by the treatments, with drought stress causing a high increase to 233.12 ng g^−1^ DW in the DS+GSH0 group (Figure 5c). Under WW conditions, the addition of 50 µM GSH reduced ABA content from 114.13 to 72.99 ng g^−1^ DW, a decrease of 36.04%. The 100 µM GSH dose (115.63 ng g^−1^ DW) did not differ significantly from the control group (Figure 5c). Conversely, under DS, GSH application effectively reduced stress-induced ABA levels, with ABA in the DS+GSH100 group decreasing by 12.55% to 203.87 ng g^−1^ DW compared to untreated drought crops (Figure 5c).

GSH applications significantly increased GA, SA, and JA levels under both irrigation conditions (Figure 5d–f). In WW plants, a 100 µM GSH treatment increased GA by 237.31% (7.35 ng g^−1^ DW), SA by 415.12% (2.88 ng g^−1^ DW), and JA by 13.34% (0.61 ng g^−1^ DW) relative to the WW+GSH0 control (Figure 5d–f). Under DS, SA (0.14 ng g^−1^ DW) and JA (0.14 ng g^−1^ DW) were lowest in the DS+GSH0 group. Under DS, 100 µM GSH significantly increased these levels, with GA 44.04%, SA 1291.59%, and JA 222.25% higher than in the DS control (Figure 5d–f).

Radar plot analysis confirms that GSH-regulated growth and defense hormones contribute to a stronger and more resilient physiological state (Figure 2e and Figure 5a–f). The extended radar profile in the DS+GSH100 group compared to the untreated DS group shows that the 100 µM GSH dose effectively balances hormones and increases drought resistance (Figure 2e and Figure 5a–f).

**Figure 6:**
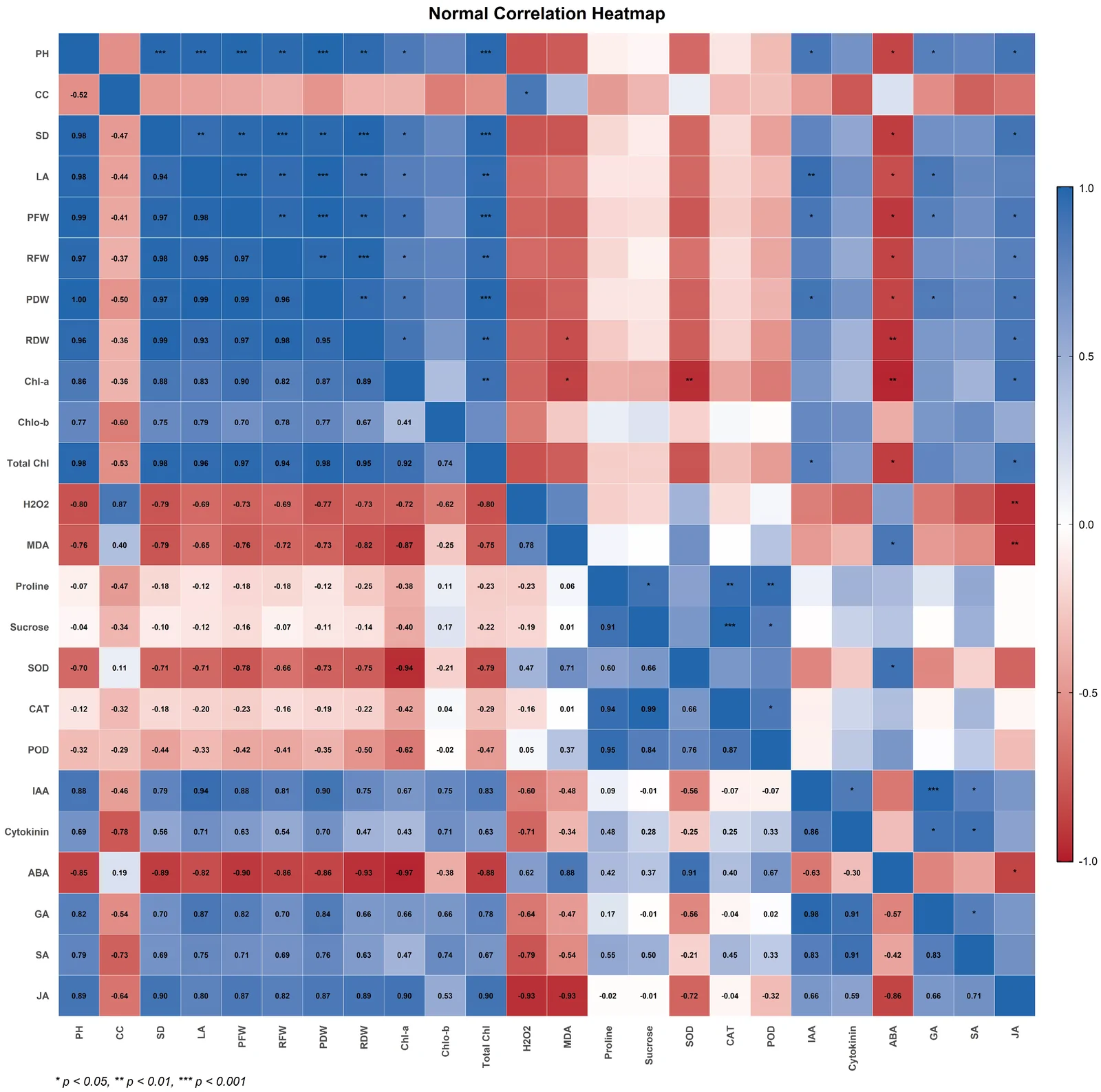
Pearson correlation heatmap showing the relationships among the measured morphological, physiological, biochemical, and phytohormone parameters in pepper plants. Correlation coefficients (r) are represented by the color scale, ranging from −1 (strong negative correlation, red) to +1 (strong positive correlation, blue), with white indicating no correlation. Asterisks indicate statistically significant correlations (* *p* < 0.05, ** *p* < 0.01, and *** *p* < 0.001). The abbreviations of the analyzed variables are provided in Table 1.

Pearson correlation analysis revealed strong and highly significant relationships between morphological, physiological, and biochemical characteristics of pepper plants (Figure 6 and Table S1). Growth and biomass parameters such as PH, SD, LA, PDW, and RDW were closely related, with correlation coefficients of at least 0.93 (*p* < 0.001). These vegetative growth characteristics also showed significant positive correlations with photosynthetic pigments, particularly total chlorophyll content (r ≥ 0.94, *p* < 0.01), and growth-promoting hormones such as IAA (r = 0.88, *p* < 0.05 for PH) and GA (r = 0.82, *p* < 0.05 for PH). In contrast, growth performance was negatively affected by the stress hormone ABA, which was inversely correlated with RDW (r = −0.93, *p* < 0.01) and PFW (r = −0.90, *p* < 0.05). Oxidative stress markers H_2_O_2_ and MDA also showed significant negative correlations with various growth and hormonal parameters, particularly with JA (r = −0.93, *p* < 0.01) (Figure 6 and Table S1). The biochemical defense system showed strong positive correlations, with osmolytes (proline and sucrose) and antioxidant enzymes (CAT and POD) showing positive correlations. Sucrose content showed a positive correlation with CAT activity (r = 0.99, *p* < 0.001), while proline was strongly associated with both CAT (r = 0.94, *p* < 0.01) and POD activities (r = 0.95, *p* < 0.01) (Figure 6 and Table S1).

**Figure 7:**
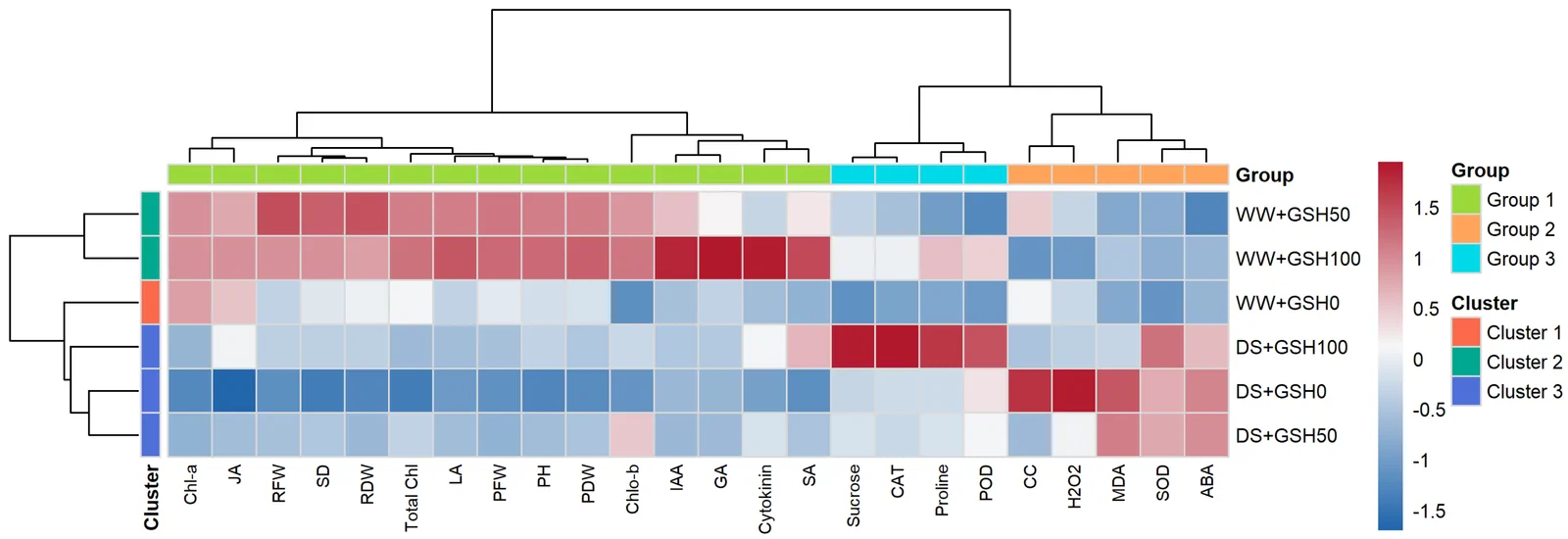
Hierarchical clustering heatmap of the measured morphological, physiological, biochemical, and phytohormone traits across glutathione (GSH) treatments under well-watered (WW) and drought stress (DS) conditions. Rows represent the experimental treatments, whereas columns represent the analyzed variables. Color intensity represents standardized values (Z-scores), ranging from −1.5 (blue, lower relative values) to +1.5 (red, higher relative values), with white indicating values close to the overall mean. The dendrograms illustrate the hierarchical clustering of both treatments and variables based on their similarity. The abbreviations of the analyzed variables are provided in Table 1.

Hierarchical clustering analysis of the treatments revealed three distinct main groups based on their overall physiological and biochemical profiles (Figure 7). Group 1 included well-irrigated treatments supplemented with exogenous GSH (WW+GSH50 and WW+GSH100). Group 2 consisted of a well-irrigated control group without GSH application (WW+GSH0). Group 3 integrated all DS treatments (DS+GSH100, DS+GSH0, and DS+GSH50). Within the DS group, the DS+GSH100 treatment formed a separate sub-cluster exhibiting a different grouping pattern compared to the DS+GSH0 and DS+GSH50 treatments (Figure 7).

The variable dendrogram grouped the measured parameters into three main clusters based on their response patterns across the treatments (Figure 7). Cluster 1 (shown in green) integrated growth-related parameters (PH, LA, PFW, PDW, RFW, RDW, SD), photosynthetic pigments (Chl-a, Chl-b, Total Chl), and growth-promoting phytohormones (IAA, GA, Cytokinin, SA, JA). Cluster 2 (shown in blue) included biochemical components associated with antioxidant defense and osmotic regulation, particularly sucrose, proline, and CAT and POD activities. Cluster 3 (shown in orange) grouped oxidative stress markers (H_2_O_2_, MDA), the stress hormone ABA, and other physiological stress indicators (SOD, CC) (Figure 7).

The heat map showed that well-irrigated groups (Group 1 and Group 2) had higher values for Cluster 1 variables and lower values for Cluster 3 variables (Figure 7). In contrast, groups under DS that lacked GSH or had low GSH content (DS+GSH0 and DS+GSH50) showed the highest intensities for oxidative damage markers and ABA (Cluster 3), while exhibiting the lowest levels for growth and pigment parameters (Cluster 1). DS+GSH100 application exhibited a different profile, characterized by high levels for Cluster 2 variables (antioxidants and osmolytes) compared to other DS groups (Figure 7).

Multivariate relationships between experimental applications and measured variables were further elucidated by three-dimensional principal component analysis with Varimax rotation (3D PCA), where the first three components (RPC1, RPC2, and RPC3) explain a significant portion (93.22%) of the total cumulative variance (Table 2). RPC1, explaining 35.57% of the variance, is primarily defined by high positive loadings for growth-promoting hormones and plant traits, particularly IAA (0.9219), GA (0.8897), cytokinin (0.8472), and leaf area (0.8172), representing the plant’s developmental vigor and hormonal optimization (Table 2). RPC2, explaining 21.75% of the variance, was characterized by strong negative loadings for defense-related biochemical parameters, including proline (−0.9692), sucrose (−0.9478), and CAT activity (−0.9665), thus reflecting the status of osmoprotective and antioxidant scavenging systems (Table 2). RPC3, explaining an additional 35.91% of the variance, highlighted the balance between structural/pigment integrity and oxidative damage. It showed high positive loadings for JA (0.8963), Chl-α (0.8168), and RDW (0.7742), in contrast to high negative loadings for MDA (−0.9753), ABA (−0.8319), and H_2_O_2_ (−0.7631) (Table 2).

**Figure 8:**
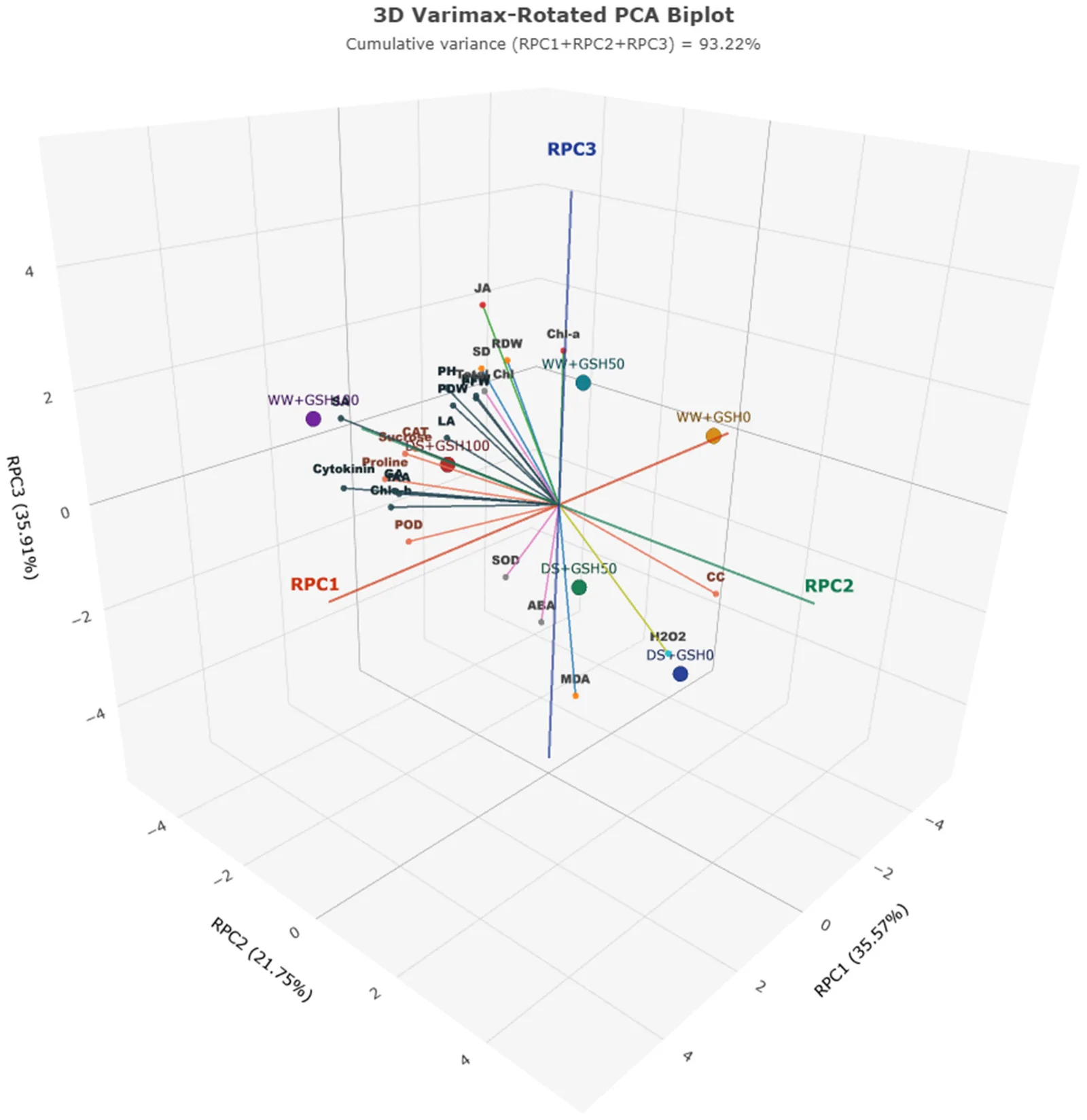
Three-dimensional Varimax-rotated principal component analysis (3D PCA) biplot showing the associations among experimental treatments and the measured morphological, physiological, biochemical, and phytohormone variables in pepper plants. Experimental treatments are represented by points, whereas arrows indicate the loading vectors of the analyzed variables. The abbreviations of the analyzed variables are provided in Table 1.

The spatial distribution of treatments in the 3D PCA biplot showed a clear separation according to WR and GSH application (Figure 8). Well-watered (WW) groups were clearly separated from DS groups. WW+GSH100 application exhibited the strongest association with the positive vectors of RPC1 and RPC3, consistent with maximum leaf area, photosynthetic pigments, and growth-promoting phytohormones (Figure 8). In contrast, untreated drought plants (DS+GSH0) were located close to the vectors of H_2_O_2_, MDA, and ABA, confirming the severe oxidative stress and growth restriction associated with this group (Figure 8). Specifically, GSH supplementation (DS+GSH100) under water deficiency caused a marked shift in the PCA space away from indicators of oxidative stress towards vectors representing antioxidant enzymes and osmolytes, demonstrating a systemic restructuring of the plant’s physiological and biochemical defense profile (Figure 8).

PCA results distinguished the experimental groups and showed that GSH-induced biological changes were dose-dependent and highly coordinated (Table 2 and Figure 8). The strong concordance of the WW+GSH100 and DS+GSH100 groups with growth-promoting and stress-reducing variables, respectively, confirms that GSH application optimizes hormonal balance and strengthens cellular defense mechanisms (Table 2 and Figure 8). This multidimensional distribution demonstrates that high loading values for growth characteristics and enzymes directly reflect the superior performance and resilience of GSH-treated plants. The isolation of the DS+GSH0 group highlights the dominance of stress markers under untreated drought conditions (Table 2 and Figure 8).

## 3. Discussion

This study investigated the effects of exogenous GSH application on water-restricted pepper seedlings and determined that GSH mitigated the reduced plant growth and development caused by water restriction. In the study, seedling height, stem diameter, and leaf area were significantly reduced in pepper seedlings. The most prominent effect of drought stress on plant growth is the suppression of growth parameters [30,31]. Due to drought, decreased cellular water potential reduces turgor pressure and inhibits cell wall elasticity and cell expansion, resulting in reduced plant height and stem thickness [29,32]. In addition, drought stress slows down activity in meristematic tissues, restricting cell number increase and leading to a decline in plant growth [33,34]. Furthermore, reduced leaf area is the most critical determinant of photosynthetic capacity and water loss in plants. In water stress situations, plants reduce leaf area to minimize water loss through transpiration. This is a defense strategy developed by the plant to conserve its water capacity [1,35]. Even mild drought conditions cause leaf cells to stop elongating in plants, leading to the formation of smaller leaves. It also accelerates leaf senescence, causing existing leaves to fall off and shrink [12,36,37]. In the study, water restriction resulted in a significant decrease in plant biomass (fresh and dry weights of plants and roots). Stress-induced stomatal closure decreases the amount of CO_2_ inside the leaf, thus slowing the rate of photosynthesis and reducing the amount of net assimilation products [1,38]. Drought reduces the activity of key enzymes responsible for carbon fixation, such as Rubisco, and causes oxidative damage to the photosynthetic apparatus, preventing the synthesis of structural components such as proteins and carbohydrates, leading to fresh and dry weight loss [12,39]. Decreased synthesis of polysaccharides such as cellulose, hemicellulose, and pectin, which make up the stem cell wall, due to drought inhibits stem cell growth and the formation of new root hairs, leading to a decrease in root biomass [1,40]. Furthermore, reduced water and nutrient uptake due to drought negatively affects root tissue development [4,41]. Morphological reductions in pepper seedlings due to water restriction result from cessation of cell expansion due to low turgor, decreased photosynthesis due to stomatal closure, and insufficient assimilates.

Water restriction decreased chlorophyll content (a, b, and total) in pepper leaves. Interestingly, SPAD values did not differ significantly among treatments, whereas extraction-based measurements showed significant changes in chlorophyll a, chlorophyll b, and total chlorophyll. This apparent discrepancy may reflect the different principles and sensitivities of the two methods. SPAD measurements provide an optical estimate of leaf greenness in intact leaves, whereas the extraction method quantitatively determines individual chlorophyll fractions on a fresh-weight basis. Thus, relatively small changes in pigment concentration that were detectable by spectrophotometric analysis may not have been sufficiently large to produce a statistically detectable change in SPAD values. One reason for the decreased chlorophyll amount due to drought is oxidative stress caused by excessive accumulation of ROS in the cells [30,39]. ROS such as superoxide radicals and hydrogen peroxide produced by stress cause lipid peroxidation in chloroplast membranes, disrupting membrane stability and leading to chlorophyll degradation [42,43]. Reduced CO_2_ influx due to stomatal closure leads to electron accumulation in photosystems and photo-oxidation of chlorophyll pigments [11,44]. Drought not only breaks down chlorophyll but also halts the synthesis of new chlorophyll molecules at different stages. This is because the activity of key enzymes in the chlorophyll biosynthesis pathway decreases due to water deficiency [1], while enzymes that accelerate the natural degradation processes of chlorophyll (chlorophyllase, pheophytinase, etc.) increase, thus accelerating pigment loss in leaves [12]. Furthermore, drought and insufficient uptake of elements such as magnesium (Mg), a key component of the chlorophyll molecule, and iron (Fe), which plays a role in its synthesis, from the soil are other reasons for decreased chlorophyll [45,46]. In this study, the decrease in chlorophyll in pepper seedlings resulted from ROS-induced membrane degradation, enzymatic chlorophyll degradation, and arrests during the synthesis phase.

Increases in H_2_O_2_, MDA, proline, sucrose, and ABA content in pepper plants under drought stress are key indicators of the plant’s response and adaptation mechanisms to oxidative damage. The fastest and most fundamental response to drought stress is ABA synthesis [7,47]. Decreased soil water potential accelerates ABA biosynthesis, transporting it through the xylem to the leaves, activating Ca channels in guard cells, and closing stomata. This defense strategy minimizes water loss through transpiration [5,30,48,49]. Drought stress triggers ROS production in cells, leading to oxidative stress [10,39]. Reduced CO_2_ fixation due to stomatal closure causes electron accumulation and the formation of superoxide (O_2_^−^) anion, which enzymes then convert into the more stable H_2_O_2_ [1,12]. Accumulation of H_2_O_2_ and hydroxyl radicals in cells disrupts cell membrane integrity and releases the breakdown product MDA. High MDA levels are an indicator of drought damage to the plant [7,46]. Proline, which accumulates in the cell cytoplasm, lowers the intracellular water potential, thus retaining water in the cell and maintaining turgor pressure [50,51], while also acting as an ROS scavenger, membrane stabilizer, and protein protector [52,53]. In drought conditions, stored starch is converted into soluble sugars such as sucrose and glucose to maintain osmotic balance in plants [1,54]. These sugars replace water molecules, stabilizing protein and membrane phospholipid structures and preventing excessive drying and cell collapse [12]. In conclusion, in peppers, increased ABA during water restriction acts as a signal for stress perception and water conservation, while the production of H_2_O_2_, another byproduct and secondary messenger of stress, increases. Stress-induced damage increases MDA, and the accumulation of proline and sucrose indicates that the plant is trying to maintain turgor under harsh conditions; these defense compounds help stabilize the cell. In this study, reduced growth in pepper seedlings results from the plant’s survival and water-conservation mechanisms. Increased ABA accumulation under drought stress has been associated with changes in the biosynthesis and regulation of growth-promoting hormones, including cytokinins, auxins, and gibberellins [3]. In the present study, the reductions in seedling height, stem diameter, and leaf area were accompanied by changes in endogenous phytohormone levels, suggesting that hormonal regulation may contribute to the observed growth responses. However, this study did not directly examine the underlying changes in hormone biosynthesis, degradation, or related gene expression.

Exogenous GSH mitigated these negative effects of drought on pepper seedlings, supporting growth under stressful conditions. In pepper plants, the improved performance observed following GSH application may be associated with coordinated changes in antioxidant defense, hormonal balance, osmotic adjustment, and morphological traits. GSH is a low-molecular-weight metabolite involved in the antioxidant defense system of plants. Previous studies have reported that exogenous GSH can modulate the expression of AsA-GSH cycle-related genes under abiotic stress [16,28]. Such molecular responses may contribute to enhanced antioxidant capacity; however, gene expression and APX/GR activities were not measured in the present study. This increase in genes is effective in raising the activity of enzymes such as APX and GR, and in clearing H_2_O_2_ and superoxide anions accumulated in cells [21,29]. Similarly, treatment with GSH in chickpeas under drought conditions increased chlorophyll synthesis and antioxidant enzyme activity [55]. It has been reported that GSH administration prevents lipid peroxidation (MDA) by reducing H_2_O_2_ levels [11,26]. In another study, the effect of GSH applied to seedlings on increasing tolerance by reducing lipid peroxidation caused by salt stress in tomato was significant [56]. Similar results have been obtained in studies showing that externally applied GSH increases tolerance to drought conditions in plants. Indeed, it has been described that GSH increased plant height, biomass, and chlorophyll content, while MDA decreased [16,57]. It reduces electrolyte leakage by protecting the cell membrane and prevents oxidative damage by preserving the structural integrity of cells [16,24]. GSH helps maintain relative water content, turgor pressure, and cell dehydration by regulating proline and total soluble sugar levels [52,58,59]. In this study, GSH application may have contributed to hormonal regulation in pepper plants. Indeed, it has been stated that GSH determines the overall growth strategy of the plant by interacting with the signaling pathways of hormones such as ABA, auxin and JA [60,61]. Endogenous GSH stimulates cell division (mitosis) in the root meristematic zone, increasing root elongation and absorption surface area [58,62]. This may allow access to deeper water during drought. It maintains plant biomass production by conserving photosynthetic pigments (chlorophyll a, b) and increasing APX/GR activities; this results in improved seedling height, fresh and dry weight, and leaf area [16,21]. Under stress conditions, GSH application increased survival rate and yield in maize, while in wheat, it strengthened antioxidant defense capacity, mitigating drought-induced damage and growth retardation [58,63]. In mung beans, it was observed to protect the plant by coordinating both the antioxidant defense system and the glyoxalase system that clears cytotoxic methylglyoxal [28]. The results we obtained from the study were along similar lines.

## 4. Materials and Methods

In the study, pepper (*Capsicum annuum* L. cv. Tatı sivri) seeds were initially sown in a peat:perlite (2:1/*v*:*v*) mixture. After approximately 30 days, seedlings were transferred to 1.5 L pots containing soil:peat:sand (1:1:1/*v*:*v*:*v*). Foliar applications were performed by spraying glutathione (GSH; Sigma-Aldrich) solutions containing 1% Tween 20 at concentrations of 50 and 100 µM onto the leaves. The GSH0 control plants were sprayed with a 1% Tween 20 solution without GSH to exclude potential effects of the surfactant. Glutathione treatments began 3 days after seedling planting and were repeated three times at one-week intervals. The study concluded after 40 days of transplanting, and morphological, physiological, and biochemical analyses were performed.

Irrigation was maintained at 100% (well-watered, WW) and 60% (drought stress, DS) of field capacity based on soil moisture measurements obtained using a Wet Sensor (Delta-T Devices, Cambridge, UK). All pots were initially brought to 100% field capacity after transplanting. Thereafter, soil moisture was regularly monitored, and water was supplied according to the measured moisture level to maintain the respective target levels. The 60% field-capacity treatment was initiated after the first GSH application. Thus, irrigation was adjusted based on soil moisture measurements rather than according to a fixed irrigation volume.

The study was conducted using a randomized plot design with a total of 108 plants, comprising three replicates, two irrigation treatments (well-watered (WW, 100%) and drought stress (DS, 60% field capacity)), three GSH doses (GSH0:0 (control), GSH50:50 µM, and GSH100:100 µM), and 6 plants per replicate. Each 1.5 L pot contained a single pepper plant.

In all plants within each replicate, measurements were taken for plant height, stem diameter, plant fresh weight, plant dry weight, root fresh weight, and root dry weight. Leaf area was measured using an area meter (CI-202 Portable Laser Leaf Area Meter, CID Bio-Science, Camas, WA, USA). Chlorophyll a, chlorophyll b, and total chlorophyll content in fresh leaf samples from each replicate were measured with a spectrophotometer at wavelengths of 645 and 663 nm and calculated as mg g^−1^ fresh weight.

For biochemical, antioxidant enzyme, and phytohormone analyses, fresh leaf samples were collected from the six plants within each experimental replicate and pooled to obtain one representative sample per replicate. Thus, three independent biological replicates (*n* = 3) were used for each treatment, and the pooled samples were stored at −80 °C until analysis. The replicate, rather than the individual plant, was considered the experimental unit for statistical analysis. To measure H_2_O_2_, samples were extracted in trichloroacetic acid (TCA) and then centrifuged at 12,000× *g* for 15 min at 4 °C. Potassium phosphate buffer (pH 7.0) and potassium iodate (KI) were then added to the supernatant, and the absorbance was measured at 390 nm. A standard curve was used to calculate the H_2_O_2_ content [64].

To determine malondialdehyde (MDA) content, samples were homogenized in TCA and reacted with thiobarbituric acid (TBA). Absorbance was measured at 450, 532, and 600 nm using a spectrophotometer (Thermo Scientific™ Multiskan™ GO Microplate Spectrophotometer, Thermo Scientific, Waltham, MA, USA), and MDA concentration was calculated using an extinction coefficient of 155 mM^−1^ cm^−1^ [65].

Proline content was measured according to the method of Bates et al. [66]. Fresh leaf samples were centrifuged in sulfosalicylic acid, and the reaction mixture was incubated with acid ninhydrin and glacial acetic acid at 95 °C for 70 min. Absorbance was measured at 520 nm, and proline concentration was determined from a calibration curve.

Sucrose content was analyzed according to the method of Liu and Huang [67]. Leaf samples were collected in ethanol, and absorbance was measured using spectrophotometry. Sucrose concentration was determined according to a standard calibration curve.

To determine the activity of antioxidant enzymes catalase (CAT), peroxidase (POD), and superoxide dismutase (SOD), fresh plant leaf samples were powdered by adding liquid nitrogen to a mortar, then centrifuged at 15,000× *g* and 4 °C for 15 min after adding cold homogenization buffer. The supernatant obtained after centrifugation was used as a source for measuring antioxidant enzyme activity [68,69]. For CAT, the decrease in absorbance occurring during the conversion of H_2_O_2_ in the medium to O_2_ and H_2_O was read at 240 nm [70], and the results were calculated as enzyme units per g of leaf (EU g leaf^−1^) [71]. For POD, the increase in absorbance caused by the colored compound, the product of the reaction with guaiacol H_2_O_2_ as the substrate, was monitored at 470 nm [68] and the results were expressed as enzyme units per leaf (EU g leaf^−1^) [72]. For SOD, it was determined spectrophotometrically at 560 nm by inhibiting the photochemical reduction in nitro blue tetrazolium, and the values were calculated as EU g leaf^−1^ [73,74].

Plant samples for hormone analysis were purified using the method first described by Kuraishi et al. [75], and hormone contents were separated using a Zorbax Eclipse-AAA C-18 column on an Agilent 1200 HPLC instrument as described by Turan et al. [76] (2014) and determined with a UV detector.

A two-way analysis of variance (ANOVA) was performed to evaluate the main effects of water regime (WR; 100% and 60% field capacity) and GSH concentration (0, 50, and 100 µM), as well as their interaction (WR × GSH). When significant differences were detected, Tukey’s HSD test was used for multiple comparisons at the 5% significance level (*p* < 0.05). Results are presented as mean ± standard error (SE). Pearson correlation analysis was performed to assess relationships among the measured parameters. In addition, principal component analysis (PCA) with Varimax rotation and hierarchical clustering analysis based on Euclidean distance and Ward’s method were performed to investigate multivariate relationships between treatments and variables. Graphical visualizations were created using the ggplot2, pheatmap, plotly, and fmsb packages in R (version 4.3.2). For each group of functional parameters, radar plots were created from treatment means using fmsb, and minimum–maximum values were normalized between treatments (0–1).

## 5. Conclusions

Water deficit markedly reduced the growth, photosynthetic performance, and physiological status of pepper seedlings by increasing ROS and altering hormone balance. Our findings show that glutathione had significant protective effects, especially at 100 µM, by enhancing antioxidant defenses and osmotic adjustment to salt-stress damage, preserving chlorophyll content, and stimulating growth-associated phytohormones while decreasing ABA (abscisic acid) accumulation. Multivariate analyses confirmed a coordinated physiological response associated with enhanced water-stress resistance and attributed it to the action of both GSH and its precursor(s). Taken together, these results indicate that exogenous glutathione is an effective biostimulant that increases drought tolerance in pepper and may be a sustainable approach to vegetable production under conditions of limited water supply. These findings should be further validated in the field, and additional studies are needed to determine how GSH functions quantitatively at the molecular level to enable crops to tolerate drought.

## Figures and Tables

**Table 1:** Mean squares from two-way analysis of variance for the effects of water regime (WR), foliar glutathione (GSH), and their interaction (WR × GSH) on growth, physiological, biochemical, and hormonal traits of pepper.

Parameter	WR	GSH	WR × GSH	Error
	*(df = 1)*	*(df = 2)*	*(df = 2)*	*(df = 12)*
PH (cm)	73.12 ***	18.86 ***	0.9914 *ns*	0.4583
CC (SPAD)	0.5689 *ns*	4.115 *ns*	2.521 *ns*	1.192
SD (cm)	1.269 ***	0.3116 ***	0.01417 *	0.00209
LA (cm^2^ plant^−1^)	40,580 ***	8755 ***	2996 ***	20.40
PFW (g plant^−1^)	54.78 ***	7.395 ***	1.388 ***	0.01809
RFW (g plant^−1^)	13.46 ***	3.939 ***	0.8146 ***	0.01167
PDW (g plant^−1^)	0.3235 ***	0.06915 ***	0.00729 *	0.00157
RDW (g plant^−1^)	0.04302 ***	0.00721 ***	0.00144 ***	0.000100
Chl-a (mg g^−1^)	5.178 ***	0.06196 *ns*	0.03026 *ns*	0.02004
Chl-b (mg g^−1^)	0.1805 ***	0.7262 ***	0.1088 ***	0.00663
Total Chl (mg g^−1^)	7.279 ***	1.204 ***	0.04790 *ns*	0.02286
H_2_O_2_ (mmol kg^−1^)	73.98 ***	50.24 ***	19.90 ***	0.1020
MDA (mmol kg^−1^)	881.0 ***	66.95 ***	168.7 ***	1.271
Proline (mmol kg^−1^)	0.00215 ***	0.00379 ***	0.0000500 *ns*	0.0000200
Sucrose (%)	1.300 ***	1.498 ***	0.3539 ***	0.00229
SOD (EU g^−1^ leaf^−1^)	56,950 ***	849.0 ***	240.6 ***	16.27
CAT (EU g^−1^ leaf^−1^)	1,497,000 ***	1,616,000 ***	397,900 ***	2800
POD (EU g^−1^ leaf^−1^)	17,080 ***	9990 ***	121.0 *ns*	66.03
IAA (ng g^−1^)	518.9 ***	197.4 ***	145.0 ***	0.04137
Cytokinin (ng g^−1^ DW)	5.655 ***	14.51 ***	4.411 ***	0.04878
ABA (ng g^−1^ DW)	65,740 ***	800.9 ***	1694 ***	3.572
GA (ng g^−1^ DW)	31.78 ***	13.87 ***	8.667 ***	0.3086
SA (ng g^−1^ DW)	2.154 ***	6.537 ***	0.09427 ***	0.00132
JA (ng g^−1^ DW)	0.3090 ***	0.05496 ***	0.02199 ***	0.000200

Values are mean squares. Significance (based on the F-test against the error term) is shown for the WR, GSH and WR × GSH sources. *** *p* < 0.001, * *p* < 0.05; *ns*, not significant. Mean squares are given to four significant figures. WR, water regime; GSH, glutathione; PH, plant height; CC, chlorophyll content (SPAD); SD, stem diameter; LA, leaf area; PFW/RFW, plant/root fresh weight; PDW/RDW, plant/root dry weight; Chl, chlorophyll; MDA, malondialdehyde; SOD, superoxide dismutase; CAT, catalase; POD, peroxidase; IAA, indole-3-acetic acid; ABA, abscisic acid; GA, gibberellic acid; SA, salicylic acid; JA, jasmonic acid.

**Table 2:** Varimax-rotated principal component loadings of the measured traits.

Variable	RPC1	RPC2	RPC3
PH	0.7244	0.0617	0.6786
CC	−0.4644	0.5311	−0.3873
SD	0.6204	0.1335	0.7444
LA	0.8172	0.1554	0.5414
PFW	0.7065	0.1911	0.6692
RFW	0.6653	0.1516	0.6587
PDW	0.7568	0.1259	0.6377
RDW	0.5525	0.1971	0.7742
Chl-a	0.4216	0.35	0.8168
Chl-b	0.8484	−0.1159	0.2046
Total Chl	0.6808	0.2083	0.6931
H_2_O_2_	−0.452	0.3134	−0.7631
MDA	−0.143	0.0267	−0.9753
Proline	0.1182	−0.9692	−0.1354
Sucrose	0.0088	−0.9478	−0.036
SOD	−0.3297	−0.606	−0.6589
CAT	−0.0746	−0.9665	−0.0386
POD	0.0569	−0.8819	−0.4393
IAA	0.9219	0.0125	0.3175
Cytokinin	0.8472	−0.3907	0.1983
ABA	−0.3577	−0.3877	−0.8319
GA	0.8897	−0.0512	0.3016
SA	0.7369	−0.5232	0.4176
JA	0.4189	−0.0582	0.8963
Eigen value	8.536	5.22	8.617
Variance (%)	35.57	21.75	35.91
Cumulative variance (%)	35.57	57.32	93.22

Abbreviations were provided in Table 1.

## Data Availability

The data supporting this study can be shared upon reasonable request.

## Supplementary Materials

The following supporting information can be downloaded at: https://www.mdpi.com/article/10.3390/plants15182831/s1, Table S1: Pearson correlation coefficients among growth, physiological, biochemical, antioxidant, and phytohormone-related traits in pepper.

## Abbreviations

The following abbreviations are used in this manuscript:

GSH	Glutathione
ROS	Reactive oxygen species
H_2_O_2_	Hydrogen peroxide
MDA	Malondialdehyde
CAT	Catalase
POD	Peroxidase
SOD	Superoxidase
ABA	Abscisic acid
IAA	Indole acetic acid
GA	Gibberellic acid
JA	Jasmonic acid
