# Supplementary material for: Multilevel Analysis of Glutathione-Induced Responses in Pepper Seedlings Under Water-Deficit Conditions
Source: Plants (Basel). 2026 Sep 16;15(18):2831. doi: 10.3390/plants15182831 (PMC13610740; doi:10.3390/plants15182831)
Supplement: Supplementary file 1 [file plants-15-02831-s001.zip › plants-4515317-supplementary.pdf]

Table S1. Pearson correlation coefficients among growth, physiological, biochemical, antioxidant, and

Variable	PH	CC	SD	LA	PFW	RFW
**PH**	-	-0.52	0.98***	0.98***	0.99***	0.97**
**CC**	-0.52	-	-0.47	-0.44	-0.41	-0.37
**SD**	0.98***	-0.47	-	0.94**	0.97**	0.98***
**LA**	0.98***	-0.44	0.94**	-	0.98***	0.95**
**PFW**	0.99***	-0.41	0.97**	0.98***	-	0.97**
**RFW**	0.97**	-0.37	0.98***	0.95**	0.97**	-
**PDW**	1.00***	-0.50	0.97**	0.99***	0.99***	0.96**
**RDW**	0.96**	-0.36	0.99***	0.93**	0.97**	0.98***
**Chl-a**	0.86*	-0.36	0.88*	0.83*	0.90*	0.82*
**Chlo-b**	0.77	-0.60	0.75	0.79	0.70	0.78
**Total Chl**	0.98***	-0.53	0.98***	0.96**	0.97***	0.94**
**H2O2**	-0.80	0.87*	-0.79	-0.69	-0.73	-0.69
**MDA**	-0.76	0.40	-0.79	-0.65	-0.76	-0.72
**Proline**	-0.07	-0.47	-0.18	-0.12	-0.18	-0.18
**Sucrose**	-0.04	-0.34	-0.10	-0.12	-0.16	-0.07
**SOD**	-0.70	0.11	-0.71	-0.71	-0.78	-0.66
**CAT**	-0.12	-0.32	-0.18	-0.20	-0.23	-0.16
**POD**	-0.32	-0.29	-0.44	-0.33	-0.42	-0.41
**IAA**	0.88*	-0.46	0.79	0.94**	0.88*	0.81
**Cytokinin**	0.69	-0.78	0.56	0.71	0.63	0.54
**ABA**	-0.85*	0.19	-0.89*	-0.82*	-0.90*	-0.86*
**GA**	0.82*	-0.54	0.70	0.87*	0.82*	0.70
**SA**	0.79	-0.73	0.69	0.75	0.71	0.69
**JA**	0.89*	-0.64	0.90*	0.80	0.87*	0.82*

***, P < 0.05;**
****, P < 0.01;**
*****, P < 0.001.**

phytohormone-related traits in pepper

PDW	RDW	Chl-a	Chlo-b	Total Chl	H2O2	MDA (mmol/l
1.00***	0.96**	0.86*	0.77	0.98***	-0.80	-0.76
-0.50	-0.36	-0.36	-0.60	-0.53	0.87*	0.40
0.97**	0.99***	0.88*	0.75	0.98***	-0.79	-0.79
0.99***	0.93**	0.83*	0.79	0.96**	-0.69	-0.65
0.99***	0.97**	0.90*	0.70	0.97***	-0.73	-0.76
0.96**	0.98***	0.82*	0.78	0.94**	-0.69	-0.72
-	0.95**	0.87*	0.77	0.98***	-0.77	-0.73
0.95**	-	0.89*	0.67	0.95**	-0.73	-0.82*
0.87*	0.89*	-	0.41	0.92**	-0.72	-0.87*
0.77	0.67	0.41	-	0.74	-0.62	-0.25
0.98***	0.95**	0.92**	0.74	-	-0.80	-0.75
-0.77	-0.73	-0.72	-0.62	-0.80	-	0.78
-0.73	-0.82*	-0.87*	-0.25	-0.75	0.78	-
-0.12	-0.25	-0.38	0.11	-0.23	-0.23	0.06
-0.11	-0.14	-0.40	0.17	-0.22	-0.19	0.01
-0.73	-0.75	-0.94**	-0.21	-0.79	0.47	0.71
-0.19	-0.22	-0.42	0.04	-0.29	-0.16	0.01
-0.35	-0.50	-0.62	-0.02	-0.47	0.05	0.37
0.90*	0.75	0.67	0.75	0.83*	-0.60	-0.48
0.70	0.47	0.43	0.71	0.63	-0.71	-0.34
-0.86*	-0.93**	-0.97**	-0.38	-0.88*	0.62	0.88*
0.84*	0.66	0.66	0.66	0.78	-0.64	-0.47
0.76	0.63	0.47	0.74	0.67	-0.79	-0.54
0.87*	0.89*	0.90*	0.53	0.90*	-0.93**	-0.93**

Proline	Sucrose	SOD	CAT	POD	IAA	Cytokinin
-0.07	-0.04	-0.70	-0.12	-0.32	0.88*	0.69
-0.47	-0.34	0.11	-0.32	-0.29	-0.46	-0.78
-0.18	-0.10	-0.71	-0.18	-0.44	0.79	0.56
-0.12	-0.12	-0.71	-0.20	-0.33	0.94**	0.71
-0.18	-0.16	-0.78	-0.23	-0.42	0.88*	0.63
-0.18	-0.07	-0.66	-0.16	-0.41	0.81	0.54
-0.12	-0.11	-0.73	-0.19	-0.35	0.90*	0.70
-0.25	-0.14	-0.75	-0.22	-0.50	0.75	0.47
-0.38	-0.40	-0.94**	-0.42	-0.62	0.67	0.43
0.11	0.17	-0.21	0.04	-0.02	0.75	0.71
-0.23	-0.22	-0.79	-0.29	-0.47	0.83*	0.63
-0.23	-0.19	0.47	-0.16	0.05	-0.60	-0.71
0.06	0.01	0.71	0.01	0.37	-0.48	-0.34
-	0.91*	0.60	0.94**	0.95**	0.09	0.48
0.91*	-	0.66	0.99***	0.84*	-0.01	0.28
0.60	0.66	-	0.66	0.76	-0.56	-0.25
0.94**	0.99***	0.66	-	0.87*	-0.07	0.25
0.95**	0.84*	0.76	0.87*	-	-0.07	0.33
0.09	-0.01	-0.56	-0.07	-0.07	-	0.86*
0.48	0.28	-0.25	0.25	0.33	0.86*	-
0.42	0.37	0.91*	0.40	0.67	-0.63	-0.30
0.17	-0.01	-0.56	-0.04	0.02	0.98***	0.91*
0.55	0.50	-0.21	0.45	0.33	0.83*	0.91*
-0.02	-0.01	-0.72	-0.04	-0.32	0.66	0.59

ABA	GA	SA	JA
-0.85*	0.82*	0.79	0.89*
0.19	-0.54	-0.73	-0.64
-0.89*	0.70	0.69	0.90*
-0.82*	0.87*	0.75	0.80
-0.90*	0.82*	0.71	0.87*
-0.86*	0.70	0.69	0.82*
-0.86*	0.84*	0.76	0.87*
-0.93**	0.66	0.63	0.89*
-0.97**	0.66	0.47	0.90*
-0.38	0.66	0.74	0.53
-0.88*	0.78	0.67	0.90*
0.62	-0.64	-0.79	-0.93**
0.88*	-0.47	-0.54	-0.93**
0.42	0.17	0.55	-0.02
0.37	-0.01	0.50	-0.01
0.91*	-0.56	-0.21	-0.72
0.40	-0.04	0.45	-0.04
0.67	0.02	0.33	-0.32
-0.63	0.98***	0.83*	0.66
-0.30	0.91*	0.91*	0.59
-	-0.57	-0.42	-0.86*
-0.57	-	0.83*	0.66
-0.42	0.83*	-	0.71
-0.86*	0.66	0.71	-
